# QSDB—a graphical Quorum Sensing Database

**DOI:** 10.1093/database/baab058

**Published:** 2021-11-26

**Authors:** Karsten Klein, Dimitar Garkov, Sina Rütschlin, Thomas Böttcher, Falk Schreiber

**Affiliations:** Department of Information and Computer Science, University of Konstanz, Universitätsstraße 10, Konstanz, Baden-Württemberg 78464, Germany; Department of Information and Computer Science, University of Konstanz, Universitätsstraße 10, Konstanz, Baden-Württemberg 78464, Germany; Department of Chemistry, Konstanz Research School Chemical Biology, Zukunftskolleg, University of Konstanz, Universitätsstraße 10, Konstanz, Baden-Württemberg 78464, Germany; Department of Chemistry, Konstanz Research School Chemical Biology, Zukunftskolleg, University of Konstanz, Universitätsstraße 10, Konstanz, Baden-Württemberg 78464, Germany; Faculty of Chemistry, Institute of Biological Chemistry and Centre for Microbiology and Environmental Systems Science, University of Vienna, UZAII, Althanstraße 14, Vienna 1090, Austria; Department of Information and Computer Science, University of Konstanz, Universitätsstraße 10, Konstanz, Baden-Württemberg 78464, Germany; Department of Information Technology, Monash University, 20 Research Way, Melbourne, Clayton, Victoria 3800, Australia

## Abstract

The human microbiome is largely shaped by the chemical interactions of its microbial members, which includes cross-talk via shared signals or quenching of the signalling of other species. Quorum sensing is a process that allows microbes to coordinate their behaviour in dependence of their population density and to adjust gene expression accordingly. We present the Quorum Sensing Database (QSDB), a comprehensive database of all published sensing and quenching relations between organisms and signalling molecules of the human microbiome, as well as an interactive web interface that allows browsing the database, provides graphical depictions of sensing mechanisms as Systems Biology Graphical Notation diagrams and links to other databases.

**Database URL**: QSDB (Quorum Sensing DataBase) is freely available via an interactive web interface and as a downloadable csv file at http://qsdb.org.

## Background

The microbiome is gaining increasing attention, and its balanced composition is of importance for human health and disease (1, 2). Chemical interactions between and within microbial populations are actively shaping the microbiome (3). Diverse microbial population behaviours as well as many important microbe–microbe and microbe–host interactions are coordinated by diffusible signalling molecules in a process known as quorum sensing (QS). QS allows microbes to coordinate their behaviour in dependence of microbial population density by regulating gene expression. This process is mediated by a variety of small molecule signals, and some species even use multiple layers of QS systems. These QS signals are typically produced in a population density-dependent manner and detected by their cognate receptors resulting in a positive feedback loop that controls gene expression (4, 5) QS regulates many important traits, including bio-film formation (6), swarming motility (7), secondary metabolites (8) and the production of major virulence factors (9).

Understanding the complex cross-talk, eavesdropping and antagonism within species of the human microbiome requires a qualitative global view of the distribution and diversity of QS systems, as well as detailed information on the ability of microbes to produce, sense or quench these signals (10).

**Figure 2.**
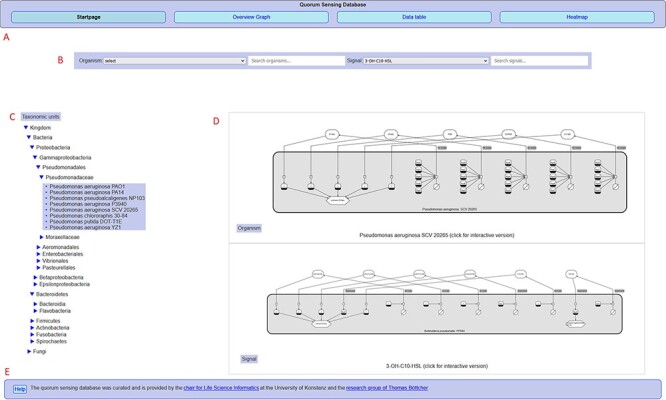
Overview of the QSDB user interface on the start page, showing (A) navigation menu to switch between the main pages, (B) organism/signal search menu, (C) taxonomic hierarchy, (D) diagram previews and (E) footer with access to help page and further information.

We developed the Quorum Sensing Database (QSDB), a database that contains manually curated, highly detailed information about QS and quorum quenching between members of the human microbiome. The interactive web interface (http://qsdb.org/, see Figure 2) supports the easy exploration of the information from overview views to single QS signals and therefore helps users to understand the complex network of interactions between organisms of the microbiome. It also allows the export of the QS data as a file in csv format.

Only few databases for QS signals have been developed so far. Quorumpeps is a database for QS peptides only (http://quorumpeps.ugent.be/) (11), while QSDB includes all classes of QS signals. Furthermore, QSDB provides greater detail such as a description of the effects of the QS molecules on the producer or other members of the gut. The database SigMol (https://bioinfo.imtech.res.in/manojk/sigmol) (12) comprises a diverse set of QS molecules; yet, it lacks the network character of QSDB with a focus on signals shared between different species of the human microbiota. This aspect of the QSDB gives the user an unprecedented opportunity to study the complex interactions within the human gut at every level of detail. In this way, it can help design future studies and guide drug development in the context of interactions within complex microbial communities.

## Data sources and content

**Figure 3.**
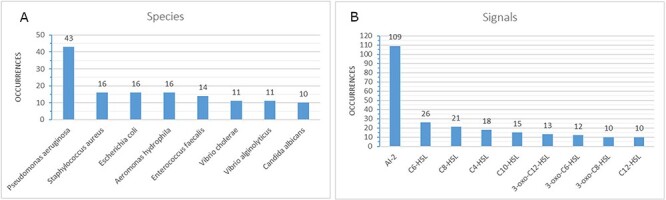
Highest numbers of occurrences for species (A) and signals (B) in the database (only values of at least 10 are shown).

**Figure 4.**
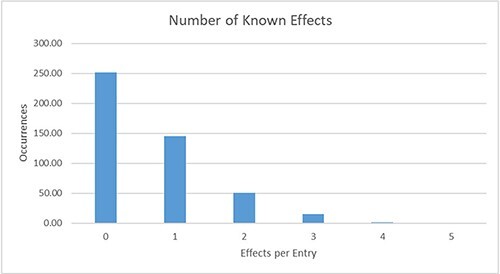
Distribution of the number of known effects per database entry. For 145 entries, exactly one effect is currently described; for 69 further entries, more than one effect is already known.

**Figure 5.**
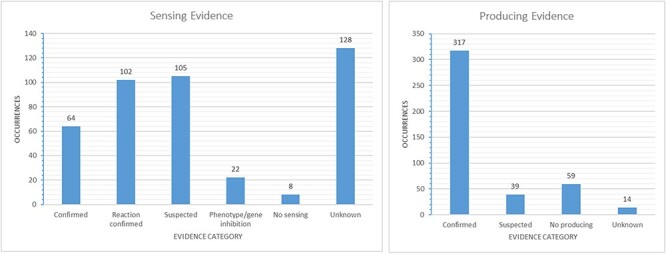
Evidence-based categories of the reported sensing and producing mechanisms: confirmed, suspected, evidence of a reaction to a signal without certainty about a receptor involved (sensing only), inhibition of a phenotype or gene through a signal (sensing only), no sensing or producing (e.g. only producing/sensing) and unknown.

Our QSDB database contains manually curated information of currently about 465 communication mechanisms (429 sensing and 36 quenching), including 123 organisms and 92 chemical signal molecules, making it the largest and most up-to-date collection of this information worldwide. QSDB stems from a collection of published QS mechanisms, and all information was extracted manually through an extensive survey of literature in the PubMed Central (PMC) and online databases. Manually curated signals, sensors, effects, type of evidence, response, sequence identity and interaction partners were added to the data collection with additional links to corresponding PubMed (13) and PubChem (14) entries. The signals that are involved in the reported mechanisms are dominated by AI-2 with 109 occurrences (see Figure 3B), whereas the species numbers are dominated by *Pseudomonas Aeruginosa* (see Figure 3A). For over 45% (214) of the known mechanisms, at least one effect is reported and stored in the database, and for 69 mechanisms, even more than one effect is known (see Figure 4). Additional information on the sensing and producing mechanisms is available, including categories such as confirmed mechanism involving a receptor, suspected sensing or producing, confirmed reaction to a signal without the mechanism being known, inhibition, producing without sensing and either unknown sensing or producing (see Figure 5).

In order to facilitate both an overview and a detailed inspection of the QS mechanisms, we opted for a graphical depiction in addition to listing the information. All information is presented using established standards in systems biology, in particular the Systems Biology Graphical Notation (SBGN) (15) for the graphical representation. As standard layout algorithms often do not compute adapted layouts for specific biological networks (16) and there are several open questions regarding good biological network visualizations (17), we implemented a simple layout algorithm tailored towards our SBGN diagrams, which produces the clickable images by placing all signalling molecules in an optimized order on the top and the species with the involved reactions on the second layer.

**Figure 1.**
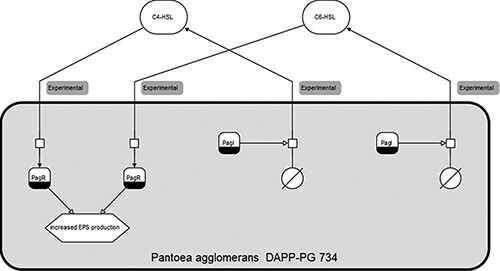
Clickable SBGN (Systems Biology Graphical Notation) diagram for Pantoea agglomerans DAPP-PG 734, with sensing and producing mechanisms for both C6-HSL and C4-HSL.

Figure 1 shows an SBGN diagram of one organism and two signalling molecules. The diagrams and accompanying data tables contain available detailed information on each sensing process, such as its known effects as well as the existing evidence for it.

## Web interface

The database is accessible via a web interface that allows detailed browsing and searching of data, user feedback and data export. For each entry, references can be found on the web interface, and direct hyperlinks lead to the corresponding PubMed online entries. A help page describes how to use the web interface.

The web interface is divided into three main pages: (i) startpage, (ii) data table page and (iii) heatmap page. A navigation area at the top of each page allows to switch between them, see Figure 2.

The startpage (i), shown in Figure 2, allows the user to select or search for entries in the database and presents static preview diagrams of sensing mechanisms as a result. Search can be performed via free text search, a taxonomic unit hierarchy or by selecting either organisms or signalling molecules from a drop-down list. Clicking on the previews leads to detailed interactive SBGN diagrams that depict the published knowledge about the QS mechanisms associated with the selected entity, with tables listing the corresponding data and showing evidence publications with links to PubMed. Entities in the diagrams, such as the signalling molecules and effects in Figure 1, are clickable with links to the respective PubChem and PubMed entries. A link leads to an additional overview diagram of the full set of signalling mechanisms in the database.

**Figure 6.**
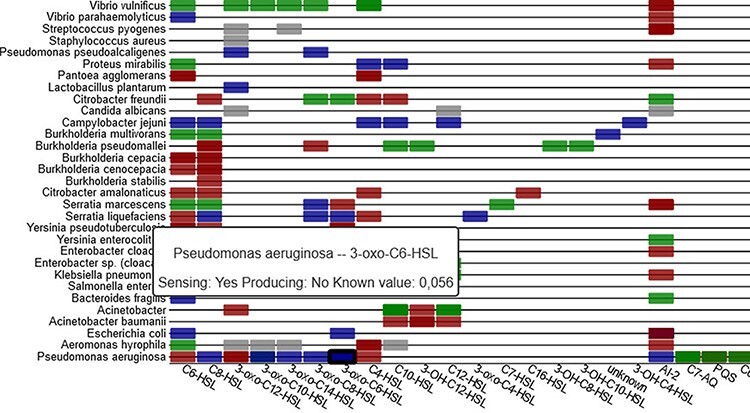
Sensing information between organisms and signal molecules is depicted in a heat-map (part shown), with colour coding representing the different classes of mechanisms (sensing, producing, inhibiting and unclear).

The data table page (ii) shows the full database in sortable table format with linked reference information and allows download of the full data set. In addition to the information on the diagram pages, it also contains known data on the relative sensing response, relative producing levels and details on the sensing/producing mechanism (e.g. ‘reacts to signal but uncertain if by receptor or other ways’). On the heatmap page (iii), all QS mechanisms from the database are graphically represented in an interactive heatmap that lists molecules on the x-axis and organisms on the y-axis (see Figure 6). The heatmap entries are clickable and lead to the corresponding primary literature on PubMed. The entries are coloured based on the sensing mechanism, and tooltips give detailed information when hovering over an entry.

### Implementation

The collected information was imported to a MariaDB database. QSDB is implemented using a standard LAMP stack architecture with MariaDB (18) as RDBMS and additionally D3 (19) for the heatmap visualization on the web server. In order to visualize the interactions, annotated networks were created for each species and signal, as well as for the complete data set. The SBGN diagrams were then created using the VANTED framework (20) with the SBGN-ED (21) Add-on. The web interface uses javascript and php to support the search functionality, data retrieval and visualization.

## Manual

The QSDB webpage (http://qsdb.org) features a help page that explains how to use the resource how to download the content and how to request additions to the database. The help page is available at https://qsdb.uni-konstanz.de/help.html.
