# Corrigendum to: QSDB—a graphical Quorum Sensing Database

**DOI:** 10.1093/database/baab071

**Published:** 2021-11-26

**Authors:** Karsten Klein, Dimitar Garkov, Sina Rütschlin, Thomas Böttcher, Falk Schreiber

**Affiliations:** Department of Information and Computer Science, University of Konstanz, Universitätsstraße 10, Konstanz, Baden-Württemberg 78464, Germany; Department of Information and Computer Science, University of Konstanz, Universitätsstraße 10, Konstanz, Baden-Württemberg 78464, Germany; Department of Chemistry, Konstanz Research School Chemical Biology, Zukunftskolleg, University of Konstanz, Universitätsstraße 10, Konstanz, Baden-Württemberg 78464, Germany; Department of Chemistry, Konstanz Research School Chemical Biology, Zukunftskolleg, University of Konstanz, Universitätsstraße 10, Konstanz, Baden-Württemberg 78464, Germany; Institute of Biological Chemistry and Centre for Microbiology and Environmental Systems Science, University of Vienna, UZAII, Althanstraße 14, Vienna 1090, Austria; Department of Information and Computer Science, University of Konstanz, Universitätsstraße 10, Konstanz, Baden-Württemberg 78464, Germany; Department of Information Technology, Monash University, 20 Research Way, Melbourne, VIC 3800, Australia

In the originally published version of this manuscript, there was an error in the Funding section. This has now been corrected online.

